# Loss of primary cilia promotes EphA2‐mediated endothelial‐to‐mesenchymal transition in the ovarian tumor microenvironment

**DOI:** 10.1002/1878-0261.70057

**Published:** 2025-05-21

**Authors:** Jin Gu Cho, Yubin Hah, Eunsik Yun, Hye In Ka, Aram Lee, Sora Han, Dawn Lee, Sung Wook Kim, Jong Hoon Park, Byung Su Kwon, Young Yang, Jongmin Kim

**Affiliations:** ^1^ Division of Biological Sciences Sookmyung Women's University Seoul Korea; ^2^ Research Institute for Women's Health Sookmyung Women's University Seoul Korea; ^3^ Department of Obstetrics and Gynecology Kyung Hee University College of Medicine, Kyung Hee University Medical Center Seoul Korea; ^4^ Department of Obstetrics and Gynecology Pusan National University School of Medicine, Biomedical Research Institute, Pusan National University Hospital Korea

**Keywords:** endothelial‐to‐mesenchymal transition (EndMT), EPH receptor A2 (*EphA2*), kinesin family protein 3a (*Kif3a*), ovarian cancer, primary cilia

## Abstract

Endothelial‐to‐mesenchymal transition (EndMT) is closely associated with tumor progression. Endothelial cells (ECs) in the tumor microenvironment (TME) use EndMT programs to facilitate tumor progression; however, the underlying mechanisms in ovarian cancer are poorly understood. Here, we describe the involvement of primary cilia in EndMT of the ovarian TME. We showed that ECs from human ovarian tumors displayed robust EndMT and impaired cilia formation, as was also observed in ECs in response to ovarian cancer cell culture‐conditioned media (OV‐CM). Notably, ECs lacking primary cilia exhibited increased OV‐CM‐induced EndMT. Vascular abnormalities, such as enhanced cell migration and vessel permeability, were observed *in vitro*. Furthermore, *in vivo* experiments using endothelial‐specific kinesin family member 3A (*Kif3a*)‐knockout mice showed enhanced EndMT in the ovarian TME. Mechanistically, we identified ephrin type‐A receptor 2 (EphA2) as a key regulator of EndMT. Upon OV‐CM treatment, *EphA2* expression increased, and depletion of *EphA2* in ECs decreased OV‐CM‐induced EndMT and vascular abnormalities. These results highlight that the loss of primary cilia and the consequent EphA2 activation are key mechanisms by which EndMT programs induce the acquisition of cancer‐associated fibroblast‐like cells in the ovarian TME, thereby promoting ovarian cancer progression.

## Graphical abstract

Loss of primary cilia in endothelial cells promotes EndMT and vascular abnormalities in the ovarian tumor microenvironment through EphA2 activation. Using human samples, *in vitro* models, and endothelial‐specific Kif3a‐knockout mice, we show that primary cilia loss drives the acquisition of cancer‐associated fibroblast‐like phenotypes, thereby facilitating ovarian cancer progression.

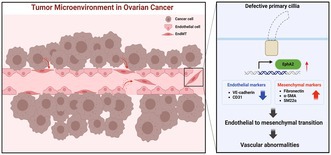

**Abbreviations**
CAF: cancer‐associated fibroblast
EC: endothelial cell
EndMT: endothelial‐to‐mesenchymal transition
EphA2: EPH receptor A2
FN: fibronectin
Kif3a: kinesin family member 3A
OV‐CM: ovarian cancer cell culture‐conditioned media
TAE: tumor‐associated endothelial cell
TME: tumor microenvironment
VE‐cadherin: vascular endothelial cadherin
α‐SMA: alpha‐smooth muscle actin

## 1 Introduction

Ovarian cancer originates in the female reproductive organs and is classified according to the cell type [1]. Because of the variety of pathologies originating from different cell types and the absence of sensible clinical signs, few patients are diagnosed with early‐stage ovarian cancer. Moreover, late‐stage and high‐grade ovarian cancer rapidly metastasizes to the omentum and surrounding tissues, resulting in a poor prognosis for most patients with ovarian cancer diagnosed at an advanced stage [2]. Currently, the most recommended standard treatment for ovarian cancer is surgical cytoreduction, followed by chemotherapy with carboplatin and paclitaxel (Taxol®) [3]. Despite a high initial sensitivity to primary treatment, most patients exhibit chemotherapy resistance, resulting in ovarian cancer recurrence and eventual death [4, 5]. Therefore, discovering novel therapeutic options via a better understanding of the basis for ovarian cancer, as well as developing new drugs to improve the efficacy of ovarian cancer treatment, is urgent.

Endothelial‐to‐mesenchymal transition (EndMT) is a process in which endothelial cells (ECs) lose some of their endothelial functions, including cell polarity and cell‐to‐cell tight junctions, while gaining mesenchymal properties, including invasiveness [6]. Therefore, during EndMT, ECs acquire mesenchymal‐specific markers, including fibronectin (FN) and alpha‐smooth muscle actin (α‐SMA), while losing endothelial‐specific markers such as vascular endothelial cadherin (VE‐cadherin) [7, 8]. EndMT was first described for its role in heart development [9], and subsequent studies have shown that EndMT plays a key role in the postnatal pathogenesis of multiple diseases such as tissue fibrosis [10], pulmonary arterial hypertension [11], and atherosclerosis [12]. Emerging evidence has shown the existence of EndMT in several types of cancers, including glioblastoma, melanoma, mammary tumors, and Kaposi sarcoma [13, 14, 15, 16]. In addition, studies have suggested that EndMT contributes to phenotypic changes with different functions in endothelial permeability and angiogenic properties, indicating that EndMT may enhance the transendothelial migration of cancer cells [6]. However, the role of EndMT in the ovarian tumor microenvironment (TME) during cancer progression remains unclear.

Primary cilia have been recently shown to play a role in regulating EndMT by modulating several key signaling pathways [17, 18]. The association between the primary cilia and EndMT was first observed during cardiac development, during which ECs in endocardial cushions, where the shear stress is high, are non‐ciliated and undergo robust EndMT in a TGF‐β‐dependent manner [19, 20]. ECs that acquire mesenchymal function migrate into the cardiac jelly to form valves [19]. Additionally, the association between primary cilia and EndMT has been implicated in various biological processes and pathologies. During heterotopic bone formation, EndMT generates endothelial‐derived MSC in response to BMP‐4, and the lack of primary cilia sensitizes ECs to undergo BMP‐dependent EndMT [17, 21]. Furthermore, endothelial‐derived fibroblasts produced by EndMT induce pulmonary fibrosis and the loss of primary cilia in ECs promotes EndMT and exacerbates pulmonary fibrosis [22]. However, the role of the primary cilia in EndMT during cancer progression remains unclear. In this study, we identified an association between primary cilia and EndMT in the ovarian TME. Interestingly, tumor‐associated ECs showed a loss of primary cilia and enhanced EndMT through EphA2 signaling, suggesting that EndMT is a key mechanism for acquiring fibroblast‐like cells and vascular abnormalities in the ovarian TME, and could be a therapeutic option for the treatment of ovarian cancer.

## 2 Materials and methods

### 2.1 Reagents and antibodies

The following antibodies were used for immunoblotting and immunofluorescence: β‐actin (#sc‐47778), GAPDH (#sc‐47724) (Santa Cruz Biotechnology, Dallas, TX, USA); Kif3a (#8507), EphA2 (#6997), p‐EphA2 (Tyr 772, #8244), Fibronectin/FN 1 (#26836), α‐Smooth Muscle Actin (α‐SMA, #19245), Transgelin 2 (SM22α, #62567), S100A4 (FSP‐1, #13018), CD31 (#3528), VE‐cadherin (#2500) (Cell signaling, Danvers, MA, USA); ARL13B (Proteintech, Chicago, IL, USA, #17711‐1‐AP); and acetylated‐α‐tubulin (#T6793) (Sigma‐Aldrich, St. Louis, MO, USA). CD31 (BD Biosciences, Franklin Lakes, NJ, USA, #550274) and cy3‐conjugated‐α‐SMA (Sigma‐Aldrich, #C6198) were used for immunohistochemistry.

### 2.2 Isolation of tumor‐associated endothelial cells (TAEs) from patients with cancer

This study was conducted using tumor tissue samples obtained from three ovarian cancer patients who underwent surgical resection at Pusan National University Hospital in Busan, Republic of Korea, between March 4, 2019, and March 31, 2020. All research procedures were carried out in full compliance with the ethical standards of the institutional and/or national research committee, as well as the 1964 Declaration of Helsinki and its subsequent amendments or equivalent ethical guidelines. The study received approval from the Institutional Review Board (IRB) of Pusan National University Hospital (approval number: 1902‐019‐076). Prior to participation, all subjects provided written informed consent after being fully informed about the use of their biological materials and clinical data for research purposes. To disassociate cancer samples into single‐cell suspensions, they were transferred to a cell culture laboratory. After finely cutting the tissue, collagenase type I (Sigma‐Aldrich, # C0130) dissolved in Hanks' Balanced Salt Solution (HBSS, WELGENE, Taipei, Taiwan, #LB‐003‐02) at a concentration of 2 mg·mL^−1^ was added to the tissue sections and incubated in a 37 °C water bath for 1 h. The sections were inverted at intervals of 5 min. After incubation, the cells were filtered using a 70‐μm cell strainer. Primary ovarian cancer cells were collected by centrifugation at 524 ***g*** for 5 min. The cell pellet was suspended and cultured in DMEM (WELGENE) with 10% heat‐inactivated fetal bovine serum (FBS) in a humidified atmosphere of 5% CO_2_ at 37 °C. Cancer‐derived single‐cell suspensions were prepared, and the cell suspensions were subjected to magnetic activating cell sorting (MACS) using anti‐CD31 antibody‐conjugated magnetic beads (Miltenyi Biotec, Bergisch Gladbach, Germany; #130‐091‐935) following the manufacturer's instructions.

### 2.3 Cell culture

Human Umbilical Vein Endothelial Cells (HUVECs; #cc‐2517; Lonza, Basel, Switzerland and Yale VBT Core, New Haven, CT, USA) and Human Microvascular Endothelial Cells (HMVEC; #cc‐2543; Lonza, Basel, Switzerland) were cultured in EBM‐2 Basal Media (Lonza, Walkersville, MD, USA; #cc‐3156) with EGM‐2 MV SingleQuots Supplement Pack (Lonza, #cc‐4147) in a humidified atmosphere of 5% CO_2_ at 37 °C. The HUVECs were used between passages 5 and 10. SK‐OV‐3 cells (RRID: CVCL_0532) were cultured in RPMI‐1640 medium (WELGENE) supplemented with 10% FBS under the same conditions. SK‐OV‐3 cells were purchased from the Korean Cell Line Bank. All cells were routinely screened for the presence of mycoplasma by MycoAlert plus kit (#LT07‐170 Lonza, Basel, Switzerland) and authenticated within the past 3 years using STR profiling.

### 2.4 Preparation of ovarian cancer cell culture‐conditioned media (OV‐CM)

To obtain ovarian cancer cell culture‐conditioned media (OV‐CM), ovarian cancer cells SK‐OV‐3 were seeded in six‐well plates at 2 × 10^5^ cells per well in RPMI with 10% FBS. After 24 h, the cells were rinsed twice with PBS, followed by replacement of the EBM‐2 medium with 0.5% FBS and incubation for 48 h. After incubation, the medium was centrifuged at 524 ***g*** for 5 min at 25 °C to remove cellular debris, and then the supernatant was collected. For the control conditioned medium, EBM‐2 medium with 0.5% FBS was incubated under the same conditions without ovarian cancer cells.

### 2.5 Immunoblot

HUVECs were lysed with RIPA buffer (20 mm Tris/HCl pH 8.0, 150 mm NaCl, 1 mm EDTA, 1% NP‐40, 1% sodium dodecyl sulfate (SDS), and a complete protease inhibitor cocktail tablet (Roche, Basel, Switzerland, #54925800)). PhosSTOP EASYpack (Roche, #04906837001) was added to analyze phosphorylated proteins. After centrifugation, cell lysates were obtained and total protein concentrations were quantified using the Pierce BCA Protein Assay Kit (Thermo Scientific, Waltham, MA, USA, #23227). Cell lysates were mixed with 5× SDS sample buffer and heated at 99 °C for 10 min. Proteins were resolved using 10–12% SDS/PAGE gels and transferred onto 0.45‐μm pore‐sized nitrocellulose membranes (GE Healthcare, Buckinghamshire, UK). The membranes were blocked with 3% bovine serum albumin (BSA) in TBS‐T buffer (150 mm NaCl, 20 mm Tris/HCl pH 8.0, and 0.05% Tween‐20) for 30 min and incubated overnight at 4 °C with specific primary antibodies. The membranes were then washed with TBS‐T and incubated with horseradish peroxidase‐conjugated antibodies appropriate for either mouse or rabbit IgG (Enzo Life Sciences, Exeter, UK) in 5% skim milk in TBS‐T at room temperature for 2 h. Proteins were visualized using an ECL kit (Bionote, Gyeonggi‐do, Korea) and detected using a Fusion Solo‐S image analyzer (Vilber Lourmat, Collegien, France).

### 2.6 Immunofluorescence

HUVECs were seeded onto 2% gelatin‐coated glass coverslips. After 24 h, the medium was changed to CM or OV‐CM, and the cells were incubated for 24 h. The cells were fixed with 4% formaldehyde (Sigma‐Aldrich) at room temperature for 15 min, washed twice with PBS, permeabilized, and blocked with 0.1% Triton X‐100 and 3% BSA at room temperature. The cells, attached to coverslips, were incubated at room temperature with the following primary antibodies for 2 h: 1 : 200 anti‐ARL13B, anti‐acetylated‐α‐tubulin. FITC (Bethyl Laboratories, Montgomery, TX, USA, #A120‐108F) or Alexa 594 (Thermo Fisher Scientific, #A21203) secondary antibodies diluted in 3% BSA were used, and the nuclei were stained with DAPI (Sigma‐Aldrich).

### 2.7 Wound‐healing assay

HUVECs were seeded in six‐well plates at a density of 2 × 10^5^ cells per well. The next day, HUVECs were transfected. After 24 h, the cells were seeded in 12‐well plates. The monolayer was manually wound using a 200‐μL micropipette tip. The cells were gently washed with PBS, and the medium was changed to CM or OV‐CM. Images were captured under a microscope after 24 h. The width of the wound was measured using the imagej software (National Institutes of Health, Bethesda, MD, USA).

### 2.8 Cell permeability assay

HUVECs pretreated with CM or OV‐CM were seeded in 24‐well transwell inserts with a 0.4 μm pore membrane (Corning Inc., Corning, NY, USA, #353024) and incubated for 48 h to form a monolayer. Cells were incubated with CM or OV‐CM. Fluorescein‐5‐isothiocyanate (FITC)‐dextran was added to the upper chamber. The concentration of FITC‐dextran transferred to the lower chamber was determined using a microplate reader, with excitation and emission wavelengths set at 485 and 535 nm, respectively.

### 2.9 Dil‐Ac‐LDL uptake assay

HUVECs cultured in six‐well plates were treated with CM or OV‐CM for 48 h. After washing with pre‐warmed PBS twice, the cells were treated with Dil‐Ac‐LDL (10 μg·mL^−1^) in serum‐free EBM‐2 media and incubated for 30 min at 37 °C. The cells were then washed twice with PBS and five times with PBS for 5 min each. Cells were fixed with 10% formaldehyde for 5 min, and nuclei were stained with DAPI for 5 min at room temperature. After staining, the cells were washed thrice for 5 min and mounted on microscope slides. Images were acquired using a fluorescence microscope.

### 2.10 Small interference RNA (siRNA)

To suppress *Kif3a* and *EphA2* expression, HUVECs were transfected with siRNA using RNAimax (Invitrogen, Carlsbad, CA, USA). The sequence for the siRNA targeting *EphA2* was as follows: si*EphA2* (5′‐UCUAAAGAAGGCACUAGAG‐3′). For *Kif3a* silencing, the siPOOL (siTools Biotech, Planegg, Germany) containing a mix of 30 different siRNAs against *Kif3a* was used. AccuTarget™ Negative Control siRNA (Bioneer, Daejeon, Korea, #SN‐1013) was used as a control.

### 2.11 RNA isolation and qRT‐PCR

Total RNA was extracted using TRIzol reagent (Takara, Shiga, Japan) according to the manufacturer's instructions. Reverse transcription was performed using the RevertAid RT Reverse Transcription Kit (Thermo Fisher Scientific). RT‐qPCR was performed using a 2× Q‐PCR Master Mix (SMOBIO, Hsinchu, Taiwan) on a QuantStudio 3 Real‐Time PCR System (Thermo Fisher Scientific). The data of Ct values were normalized to that of β‐actin. The primers used for RT‐PCR were as follows: *Kif3a* forward (5′‐TGAGGAGAGTCTGCGTCAGT‐3′) and reverse (5′‐CTTTGCAGAACGCTTTCTTCTCC‐3′), and *EphA2* forward (5′‐AGCTGGCATGAAGTACCTGG‐3′) and reverse (5′‐GTCAGACACCTTGCAGACCA‐3′).

### 2.12 Mice

All animals were housed in ventilated cages inside pathogen‐free animal rooms with controlled temperature (22–24 °C), humidity (55–60%), and a 12 h light and 12 h dark cycle. The mice received *ad libitum* access to water and a standard chow diet (65% carbohydrate, 11% fat, 24% protein). All animal procedures were approved and performed under the supervision of the Institutional Animal Care and Use Committee (IACUC) at Sookmyung Women's University of Korea (SMWU‐IACUC‐2106‐009‐01). Endothelial‐specific *Kif3a* knockout mice were generated by breeding *Kif3a* floxed mice (Kif3a^tm2Gsn^, MGI ID: 2386464) and Cdh5‐Cre/ERT2 mice. PCR was performed on the genomic DNA isolated from the tail to confirm the floxed *Kif3a* construct with primers previously described [23, 24]. The following primers designed by Prof Goo Taeg Oh (Ewha Women's University) were used to confirm the Cdh5‐cre recombinase construct: forward 5′‐GAGCCACTCGTTCCATAGGACAG‐3′ and reverse 5′‐ATTGCTGTCACTTGGTCGTGGC‐3′. *Kif3a* floxed mice were kindly provided by Prof Hyuk Wan Ko (Yonsei University), and Cdh5‐Cre/ERT2 mice were kindly provided by Prof Goo Taeg Oh (Ewha Women's University). Generated endothelial cell‐specific *Kif3a* gene knockout mice were used as 6‐week‐old female mice in all experiments.

### 2.13 Mouse tumorigenesis experiments

To induce ovarian tumor formation, 5 × 10^6^ ID8 cells were suspended in 100 μL of a 1 : 1 mix of matrigel (BD Biosciences, Bedford, MA, USA) and subcutaneously injected into the right flank of 6‐week‐old female C57BL/6 mice using a kovax‐syringe (26.5 gauge needle). The mice were sacrificed after 8 weeks.

### 2.14 Immunohistochemistry

Tumor tissues were embedded in optimal cutting temperature (OCT) compound (#3801480, Leica Biosystems, Wetzlar, Germany). To visualize primary cilia and EndMT, frozen tumor tissues were sectioned at 6 μm, mounted onto glass, and dried at room temperature for 2 h. The sections were fixed with 4% formaldehyde for 15 min at room temperature and washed thrice with PBS. After blocking, the sections were permeabilized in 0.1% Triton X‐100 in PBS for 15 min, washed thrice with PBS, and blocked with 0.1% BSA in PBS for 30 min at room temperature. To confirm EndMT, sections were incubated with the following primary antibodies overnight at 4 °C: 1 : 100 anti‐CD31, 1 : 200 anti‐cy3‐conjugated‐anti‐α‐SMA. FITC secondary antibodies diluted in 0.1% BSA were used, and the nuclei were stained with DAPI. Images were acquired using a confocal microscope (LSM700, Zeiss, Oberkochen, Germany) at the Chronic and Metabolic Diseases Research Center of Sookmyung Women's University.

### 2.15 Statistical analysis

For multiple comparisons, one‐way factorial analysis of variance was used, and for the wound‐healing assay, two‐way analysis of variance was used. Individual group mean differences were analyzed using two‐tailed Student's *t* test, Tukey's test, and Dunnett's test. Values of *P < 0.05, **P < 0.01, and ***P < 0.001 were considered benchmarks for significant differences. All statistical analyses were performed using graphpad prism version 8.0 for Windows (GraphPad Software, San Diego, CA, USA).

## 3 Results

### 3.1 Tumor‐associated endothelial cells in human ovarian tumors display robust EndMT and impaired cilia formation

**Fig. 1:**
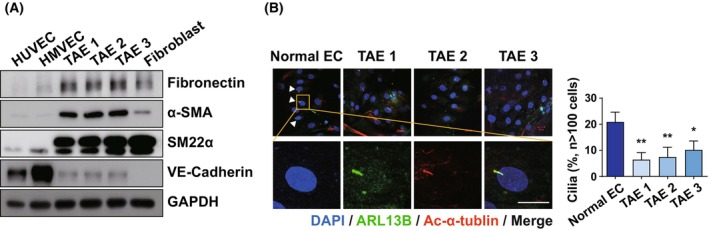
Robust EndMT and impaired primary cilia formation in Ovarian TAEs. (A) Protein expression of the endothelial markers and the mesenchymal markers in ovarian TAEs. TAEs were isolated from cell suspensions using magnetically activated cell sorting using CD31 antibody‐based magnetic cell sorting. Cell lysates were analyzed by immunoblot and GAPDH was used as the loading control. TAEs were isolated from three independent human ovarian tumor samples (*n* = 3). (B) Immunofluorescence images of primary cilia in normal ECs and ovarian TAEs. Primary cilia on the cells were immunostained with ARL13B (green), and acetylated‐α‐tubulin (red). Nuclei were stained with DAPI. The arrows indicate the primary cilia. Values represent the mean ± SD from three independent experiments. The percentage of ciliated cells was evaluated (*n* > 100 cells per condition); **P < 0.01, *P < 0.05, by one‐way ANOVA with Dunnett's test. *n* = 3 (Scale bar = 20 μm). (ECs, Endothelial cells; EndMT, Endothelial‐to‐mesenchymal transition; FN, Fibronectin; HMVEC, Human microvascular endothelial cells; HUVEC, Human umbilical vein endothelial cells; SM22α, smooth muscle protein 22‐alpha; TAE, Tumor‐associated endothelial cell; α‐SMA, alpha‐smooth muscle actin).

As growing evidence indicates the existence of EndMT in various fibrotic tissues and TME [25, 26, 27], we evaluated whether EndMT occurs in human ovarian tumor tissues. First, CD31‐positive ECs were isolated from human ovarian tumors to evaluate the presence of EndMT. Using an assay determining acetylated LDL (Ac‐LDL) uptake (a property specific to ECs), we showed that the cells were not contaminated with other cell types (not shown). Unlike normal ECs, human ovarian tumor‐associated ECs (TAEs) showed a significant increase in the levels of mesenchymal markers such as fibronectin (FN), alpha‐smooth muscle actin (α–SMA), and smooth muscle protein 22‐alpha (SM22α), whereas the expression level of the endothelial marker VE‐cadherin was reduced in ovarian TAEs (Fig. 1A). These results suggest that ovarian TAEs undergo robust EndMT and represent a type of cancer‐associated fibroblasts (CAFs) in the ovarian TME. Given that primary cilia are closely associated with EndMT during cardiac development [28], we next examined the formation of primary cilia in ovarian TAEs, where EndMT is robust. Interestingly, primary cilia formation was significantly decreased by 60% compared with that in normal ECs (Fig. 1B). Collectively, these results suggest that EndMT may be involved in impaired ciliary formation during ovarian tumor progression, and that the association between EndMT and primary cilia in the ovarian TME needs to be further investigated.

### 3.2 Endothelial cells lacking primary cilia show increased EndMT *in vitro* and *in vivo*

**Fig. 2:**
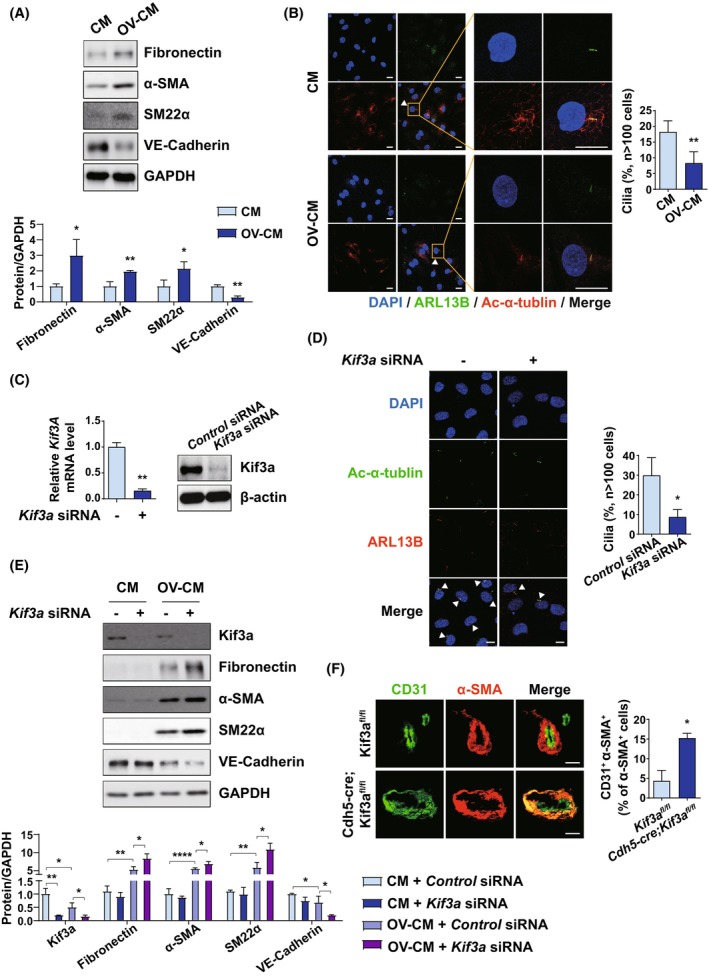
Loss of primary cilia shows increased EndMT. (A, B) HUVECs were treated with CM or OV‐CM for 24 h. (A) Protein expression of the endothelial markers and the mesenchymal markers. Cell lysates were analyzed by immunoblot and GAPDH was used as the loading control. Representative results from three independent experiments are shown. (B) Immunofluorescence images of primary cilia. Primary cilia on the cells were immunostained with ARL13B (green) and acetylated‐α‐tubulin (red). Nuclei were stained with DAPI. The arrows indicate the primary cilia. Values represent the mean ± SD from four independent experiments. The percentage of ciliated cells was determined (*n* > 100 cells per condition); **P < 0.01, by 2‐tailed student's *t* test. *n* = 4 (Scale bar = 20 μm). (C) Quantification of *Kif3a* expression in HUVECs. HUVECs were transfected with si*Kif3A* 1 ng·L^−1^ for 48 h, and siGFP was used as a negative control. The quantification was examined by RT‐qPCR (left) and immunoblot (right). mRNA and protein levels were normalized by β‐Actin; **P < 0.01, by two‐tailed student's *t* test. *n* = 3 (D) Immunofluorescence images of primary cilia in HUVECs transfected with 1 ng·L^−1^ si*Kif3A*. Primary cilia on the cells were immunostained with ARL13B (red) and acetylated‐α‐tubulin (green). Nuclei were stained with DAPI. Values represent the mean ± SD from three independent experiments. The arrows indicate the primary cilia; *P < 0.05, by two‐tailed student's *t* test. *n* = 3 (Scale bar = 20 μm) (E) Protein expression of the endothelial markers and the mesenchymal markers. HUVECs were transfected with si*Kif3A* 1 ng·L^−1^ for 48 h and treated with OV‐CM for 24 h. Cell lysates were analyzed by Immunoblot. Values represent the mean ± SD from three independent experiments. *P < 0.05, **P < 0.01, ****P < 0.0001, by 2‐tailed student's *t* test. (F) Immunofluorescence analysis of ID8 tumor sections. Tumors were sectioned at 6 μm and immunostained with CD31 (green) and α‐SMA (red). Nuclei were stained with DAPI. Values represent the mean ± SD from three independent experiments. Representative data are shown from four mice; *P < 0.05, by two‐tailed student's *t* test. (Scale bar = 20 μm). (CM, control media; EndMT, Endothelial‐to‐mesenchymal transition; FN, Fibronectin; HUVEC, Human umbilical vein endothelial cells; Kif3a, Kinesin Family Member 3A; OV‐CM, Ovarian cancer cell culture‐conditioned media; SM22α, smooth muscle protein 22‐alpha; α‐SMA, alpha‐smooth muscle Actin).

To investigate the association between EndMT and primary cilia in the ovarian TME, HUVECs were treated with ovarian cancer cell‐conditioned medium (OV‐CM) to mimic the ovarian TME and the effects of OV‐CM on EndMT were examined. OV‐CM simulates the paracrine signaling interactions between ovarian cancer cells and ECs within the TME in a controlled environment. Ovarian cancer cells secrete a variety of factors that drive cancer progression, including the induction of EndMT and impairment of primary cilia in surrounding cells. By treating ECs with OV‐CM, these interactions can be replicated *in vitro*, enabling the investigation of how specific secreted factors influence EC behavior. This approach provides a valuable model for studying the mechanisms by which the ovarian TME, through secreted factors, drives pathological processes such as EndMT and ciliary impairment. The treatment with OV‐CM increased mesenchymal markers, including FN, α–SMA, and SM22α, while reducing the levels of endothelial markers VE‐cadherin (Fig. 2A). Next, we examined whether ECs displayed impaired cilia formation following treatment with OV‐CM. We found that the proportion of ciliated cells was significantly reduced in OV‐CM‐treated HUVECs compared to that in control HUVECs (Fig. 2B). These results are consistent with robust EndMT and impaired cilia formation in ovarian TAEs and suggest that the secretome of ovarian cancer cells is sufficient to induce EndMT and impaired cilia formation in a heterogeneous TME.

To further investigate the association between EndMT and primary cilia in response to OV‐CM, kinesin family member 3a (*Kif3a*), an essential gene for cilia formation and maintenance [29], was inhibited using small interfering RNA (siRNA). Subsequently, EndMT was evaluated upon OV‐CM treatment. The successful knockdown of *Kif3a* expression was confirmed by RT‐qPCR and immunoblotting (Fig. 2C). As expected, *Kif3a* depletion dysregulated primary cilia formation in HUVECs (Fig. 2D).

Next, we evaluated whether knockdown of *Kif3a* in normal HUVECs affected OV‐CM‐induced EndMT. Consistently, normal HUVECs treated with OV‐CM showed a significant increase in mesenchymal marker levels and a decrease in endothelial marker levels. In addition, OV‐CM‐treated HUVECs showed decreased levels of Kif3a, suggesting that OV‐CM downregulates primary cilia formation by suppressing the expression of ciliogenesis‐related genes, such as Kif3a. Moreover, the expression of mesenchymal markers was substantially higher in *Kif3a*‐depleted HUVECs than in Kif3a‐intact HUVECs treated with OV‐CM, demonstrating that the absence of primary cilia accelerated OV‐CM‐induced EndMT (Fig. 2E).

To further validate whether the lack of primary cilia was associated with an increase in EndMT *in vivo*, we generated EC‐specific *Kif3a* KO mice *Cdh5‐Cre*; *Kif3a* ^*fl/fl*^ by breeding *Kif3a* ^*fl/fl*^ mice with mice expressing *Cre* under the EC‐specific promoter *Cdh5*. Genomic PCR analysis of *Cdh5‐Cre*; *Kif3a* ^*fl/fl*^ mice revealed that vascular‐rich tissues, including the kidney, lungs, and liver, were well targeted (Fig. S1). *Cdh5‐Cre*; *Kif3a* ^*fl/fl*^ was injected into the mouse ovarian cancer cell line ID8 to generate tumors. Ovarian tumor sections were immunostained for CD31, an endothelial marker, and α‐SMA, a mesenchymal marker, to determine whether primary cilia were associated with EndMT in the TME. The vascular region within the tumor tissue from *Cdh5‐Cre*; *Kif3a* ^*fl/fl*^ mice showed higher colocalization of α‐SMA and CD31, indicating that the occurrence of EndMT in *Cdh5‐Cre*; *Kif3a* ^*fl/fl*^ mice was higher than in *Kif3a* ^*fl/fl*^ mice (Fig. 2F). These data suggest that the loss of primary cilia enhances EndMT in the ovarian TME both *in vivo* and *in vitro*.

### 3.3 OV‐CM‐induced EndMT promotes vascular abnormality *in vitro*

**Fig. 3:**
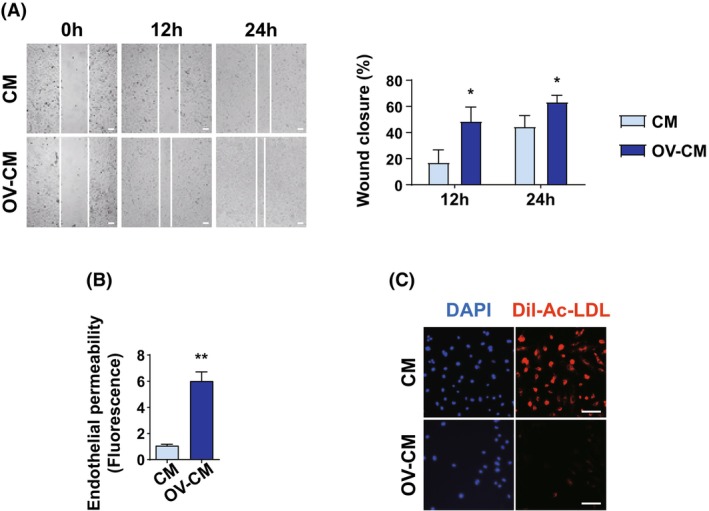
EndMT induces vascular abnormality *in vitro*. (A) Migration assay in HUVECs treated with OV‐CM. The migration of HUVECs was measured using a wound‐healing assay. Images were taken every 12 h after scratch for 24 h in the presence of OV‐CM. Values represent the mean ± SD from three independent experiments. Representative images of *n* = 3 were shown; *P < 0.05, by two‐tailed student's *t* test. (Scale bar = 200 μm) (B) Cell permeability assay in HUVECs. The permeability of an EC monolayer was investigated by measuring the fluorescence of FITC‐dextran diffusing over a transwell membrane. Values represent the mean ± SD from three independent experiments. **P < 0.01, by two‐tailed student's *t* test. *n* = 3 (C) Dil‐Ac‐LDL uptake assay in HUVECs. HUVECs were incubated with Dil‐Ac‐LDL 10 μg·mL^−1^. Nuclei were stained with DAPI (Scale bar = 100 μm). Representative images of *n* = 3 were shown. (CM, control media; EndMT, Endothelial‐to‐mesenchymal transition; HUVEC, Human umbilical vein endothelial cells; OV‐CM, Ovarian cancer cell culture‐conditioned media).

When ECs undergo EndMT, they lose cell‐to‐cell adherens junctions, resulting in increased migration ability and endothelial permeability [30]. The loss of adherens junctions is caused by a decrease in the expression of VE‐cadherin, an endothelial‐specific adhesion molecule [31]. Indeed, ECs treated with OV‐CM showed enhanced migration (Fig. 3A). Next, to determine endothelial permeability, HUVECs were plated onto the upper wells of a transwell 1 day before treatment with FITC‐dextran, and the level of FITC‐dextran in the bottom well was measured. The FITC‐dextran strongly penetrated the OV‐CM‐treated endothelial monolayer (Fig. 3B). In contrast, ECs take up acetylated low‐density lipoprotein (1,1′‐dioctadecyl–3,3,3′,3′‐tetramethyl‐indocarbocyanine perchlorate; Dil‐Ac‐LDL) [32], but EndMT reduces this trait in ECs [33]. Notably, OV‐CM‐treated ECs showed a significant decrease in the uptake of Dil‐Ac‐LDL (Fig. 3C); however, the cells partially retained the functions required for the uptake of Dil‐Ac‐LDL in vascular ECs. These results indicate that OV‐CM‐treated ECs partially reduced their endothelial‐specific functions and induced their transition into fibroblast‐like cells via EndMT.

### 3.4 EphA2 is a novel key regulator of OV‐CM‐induced EndMT

**Fig. 4:**
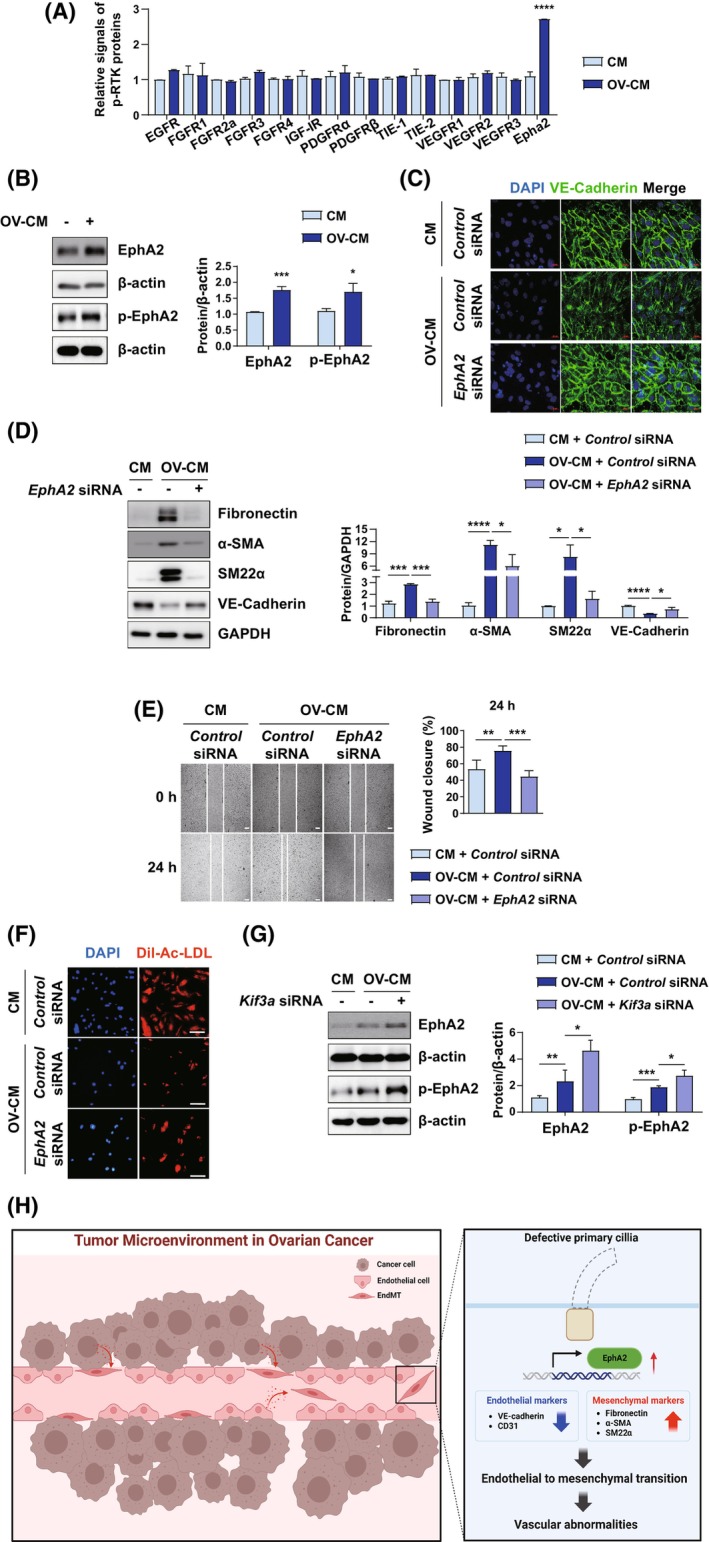
EphA2 Is a key regulator of EndMT. (A, B) HUVECs were treated with CM or OV‐CM (A) RTK assay in HUVECs. HUVECs were treated for 72 h and cell lysates were analyzed using a phosphoreceptor tyrosine kinase array. Values represent the mean ± SD from three independent experiments. ****P < 0.0001, two‐way ANOVA with Bonferroni's test. (B) Protein expression of EphA2 and p‐EphA2 at Tyr^772^. HUVECs were treated with OV‐CM or CM for 24 h and cell lysates were analyzed by Immunoblot. Values represent the mean ± SD from three independent experiments. *P < 0.05, ***P < 0.001, by 2‐tailed student's *t* test. (C, D) HUVECs transfected with si*EphA2* were treated with OV‐CM for 24 h. (C) Immunofluorescence analysis in cells with an anti–VE‐cadherin antibody. Representative images of *n* = 3 were shown (Scale bar = 40 μm). (D) Protein expression of the endothelial markers and the mesenchymal markers. Cell lysates were analyzed by immunoblot and β‐Actin was used as the loading control. Values represent the mean ± SD from three independent experiments. *P < 0.05, ***P < 0.001, ****P < 0.0001, by 2‐tailed student's *t* test. (E) Migration assay in HUVECs transfected with si*EphA2*. The migration of *EphA2* depleted HUVECs was measured using a wound‐healing assay. Images were taken after 24 h of scratch in the presence of OV‐CM. Values represent the mean ± SD from three independent experiments. Representative images of *n* = 3 were shown; **P < 0.001, ***P < 0.0001, by two‐way ANOVA with Tukey's test. (Scale bar = 200 μm) (F) Dil‐Ac‐LDL uptake assay in HUVECs. HUVECs were transfected with si*EphA2* 20 nm for 48 h and treated with OV‐CM for 24 h. *EphA2* depleted HUVECs were incubated with Dil‐Ac‐LDL 10 μg·mL^−1^. Nuclei were stained with DAPI (blue). Representative images of *n* = 3 were shown (Scale bar = 100 μm). (G) Expression of EphA2 in *Kif3a*‐depleted HUVECs treated with OV‐CM. HUVECs transfected with si*EphA2* were treated with OV‐CM for 24 h. Cell lysates were analyzed by Immunoblot and β‐Actin was used as the loading control. Values represent the mean ± SD from three independent experiments. *P < 0.05, **P < 0.01, ***P < 0.001, by 2‐tailed student's *t* test. (H) Schematic summarizing the relationship between EphA2 and primary cilia in EndMT. (CM, control media; EndMT, Endothelial‐to‐mesenchymal transition; EphA2, EPH Receptor A2; FN, Fibronectin; HUVEC, Human umbilical vein endothelial cells; Kif3a, Kinesin Family Member 3A; OV‐CM, Ovarian cancer cell culture‐conditioned media; SM22α, smooth muscle protein 22‐alpha; α‐SMA, alpha‐smooth muscle Actin).

Next, we explored the molecular mechanisms underlying the induction of EndMT by OV‐CM. Given our finding that soluble factors contained in OV‐CM induce vascular abnormalities via EndMT, we next investigated the surface receptors on ECs that regulate EndMT. Using a phosphoreceptor tyrosine kinase (phospho‐RTK) array assay, we identified that EphA2 was selectively activated when ECs were exposed to OV‐CM (Fig. 4A). Consistently, the phosphorylation of EphA2 on Tyr^722^, which is essential for kinase activity, was induced. Interestingly, the expression of EphA2 was also increased in OV‐CM‐treated ECs (Fig. 4B). To determine whether EphA2 is essential for OV‐CM‐induced EndMT, the most efficient *EphA2* siRNA was selected to deplete *EphA2* among the three *EphA2* siRNAs by immunoblotting (Fig. S2). Immunofluorescence analysis showed that OV‐CM treatment inhibited and reduced VE‐cadherin expression, leading to weaker adherent junctions, which subsequently increased vascular permeability. The effects of OV‐CM treatment were abrogated by concurrent transfection with *EphA2* siRNA (Fig. 4C). In addition, *EphA2* knockdown abrogated the expression of OV‐CM‐induced mesenchymal markers and OV‐CM‐reduced endothelial markers (Fig. 4D), suggesting that EphA2 is required for EndMT. Consistent with the important role of EphA2 in EndMT, *EphA2* knockdown reversed the abnormal migration and Dil‐Ac‐LDL uptake induced by OV‐CM treatment (Fig. 4E,F). These data indicated that EphA2 is a key regulator of OV‐CM‐induced vascular abnormalities via EndMT.

Finally, to determine whether the expression of EphA2 depends on the presence of primary cilia, the expression of OV‐CM‐induced EphA2 was investigated in *Kif3a*‐depleted ECs. Interestingly, the expression of OV‐CM‐induced EphA2 was further enhanced in *Kif3a*‐depleted ECs (Fig. 4G). Since OV‐CM reduces the number of ciliated cells and enhances EphA2 expression, this suggests that the absence of primary cilia probably enhances the expression of EphA2, leading to EndMT.

## 4 Discussion

Studies have shown that when ECs acquire mesenchymal cell‐like properties in the TME of several types of cancer, ECs proliferation, and migration are increased, promoting angiogenesis, cancer progression, and dissemination [34]. Moreover, EndMT promotes cytoskeletal remodeling and immune infiltration in the TME, affecting chemoresistance [7, 26]. Moreover, it is the major source of CAFs in the TME, contributing to cancer progression and metastasis [16]. Given these findings, targeting EndMT could be a novel and promising treatment strategy for cancer by regulating the TME, which plays a significant role in the tumorigenesis of various cancers. Several studies have shown that the secretome of ovarian cancer cells stimulates the transition of normal fibroblasts and adipose‐derived stem cells into pathogenic CAFs [35, 36]. However, the role of EndMT in ovarian cancer progression has not yet been studied, and little is known about whether ECs in the ovarian TME undergo EndMT or whether EndMT is the origin of CAFs. In this study, we revealed that EndMT contributes to vascular abnormalities, including enhanced migration and permeability, by the acquisition of a fibroblast phenotype in the ovarian TME.

EndMT induces vascular abnormalities through the loss of endothelial integrity and the acquisition of mesenchymal‐like features [37]. Treatment of ECs with OV‐CM caused a decrease in the expression of adhesion molecules, including VE‐cadherin, which led to an increase in endothelial permeability. This disruption is consistent with the process of cancer cell infiltration into both the intra‐ and extravasation, representing the initial stage of metastasis [38]. During cancer progression, EndMT is associated with angiogenesis, a key hallmark of cancer [39]. Moreover, previous studies have shown that EndMT drives aberrant vascularization and chemoresistance by promoting EC plasticity [14, 40]. Therefore, EndMT suppression may be an effective therapeutic strategy to inhibit cancer progression, metastasis, and drug resistance.

In addition, our findings are the first to show that primary cilia are closely associated with EndMT in the ovarian TME. Ovarian TAEs exhibit ciliary defects, and the loss of primary cilia in ECs enhances ovarian cancer‐induced EndMT, suggesting that the loss of primary cilia enhances the transition of ECs to CAFs through EndMT. Collectively, primary cilia are novel regulators of EndMT in the ovarian TME. However, primary cilia and the EndMT process have a complex relationship. Notably, the loss of primary cilia can be a consequence of EndMT, which can lead to endothelial dysfunction, thereby contributing to tumor progression. Therefore, future studies are required to clarify the effect of increased EndMT on primary cilia in the TME. Additionally, further studies are required to investigate the mechanisms underlying primary cilia‐mediated regulation of EndMT. These are crucial for developing novel therapies for cancers. Exploring the roles of additional ciliary proteins, such as IFT88, ARL13B, and polycystins, will enhance our understanding of the complex interplay between primary cilia and the EndMT process.

EndMT is regulated by a common set of transcriptional effectors driven by cooperation between different signaling pathways, including Wnt, TGF‐β, and cytokines [41]. In epithelial cells, primary cilia inhibit the Wnt pathway [42]. The ciliary protein, Inversin, is involved in the inhibition of the canonical Wnt pathway via the degradation of Disheveled (Dvl), while the loss of primary cilia causes the activation of Wnt by the cytoplasmic accumulation of Dvl via the depletion of Inversin [43, 44]. Moreover, in the absence of primary cilia, jouberin is released into the cytoplasm and activates the Wnt signaling pathway [45]. As the Wnt signaling pathway is coordinated in EndMT and primary cilia are also associated with the Wnt signaling pathway, it is conceivable that the loss of primary cilia enhances EndMT.

This study reveals that EphA2 is a novel and crucial regulator of EndMT in the ovarian TME. It has been reported that EphA2 acts as a receptor for Wnt ligands and that treatment with Wnt enhances the EphA2‐Dvl2/Axin1 interaction, leading to destabilization of the β‐catenin destruction complex and promoting the nuclear accumulation of β‐catenin. In the nucleus, β‐catenin activates c‐MYC transcription, and c‐MYC binds to the *EphA2* promoter, enhancing the expression of EphA2. Therefore, an EphA2‐mediated feed‐forward loop to propagate Wnt signaling is completed [46]. Non‐ciliated ECs show increased EphA2 expression, and loss of EphA2 inhibits OV‐CM‐induced EndMT. Primary cilia act as negative regulators of OV‐CM‐induced EndMT. We identified EphA2 as a molecular link between primary cilia and EndMT.

One of the most significant clinical challenges in treating ovarian cancer is platinum resistance, whereby tumors no longer respond to platinum‐based chemotherapies, such as cisplatin or carboplatin, which are the first‐line treatments [47, 48, 49]. Increasing evidence suggests that CAFs play a pivotal role in the development of platinum resistance by creating a protective microenvironment that supports tumor survival [50], reduces drug efficacy [51, 52], and enhances the tumor's ability to evade apoptosis [53]. CAFs secrete various factors, including cytokines and growth factors, that contribute to drug resistance and tumor recurrence, leading to poor clinical outcomes [54, 55, 56]. As a result, platinum‐resistant ovarian cancer is often associated with a worse prognosis and limited therapeutic options [57].

Although CAFs originate from various cell types, including resident fibroblasts [58], bone marrow‐derived mesenchymal stem cells (BM‐MSCs) [59] and smooth muscle cells [60], several studies have shown that CAFs in the TME originate from ECs via the EndMT process [16]. Our data showed that TAEs from human ovarian tumors display a robust EndMT and highly express α‐SMA, the indicator of activated CAFs during EndMT [61]. This suggests that EndMT enhances the transition from ovarian TAEs to CAFs. In addition to α‐SMA, there are other biomarkers of CAFs, including fibroblast activation protein‐α, fibroblast‐specific protein‐1, and platelet‐derived growth factor α receptors, which are also altered in ovarian cancer [62]. While further studies are required to determine the expression levels of these biomarkers, our findings indicate that EndMT is also a source of CAF formation in the ovarian TME. Moreover, our findings show that EphA2 inhibition is a promising approach to overcoming these limitations by targeting EndMT‐driven CAF formation. Inhibiting EphA2 could reduce the CAF population, sensitize platinum‐resistant tumors to chemotherapy, and improve drug delivery by normalizing the tumor vasculature. This would reduce abnormal vascular permeability, limit metastasis, and slow tumor progression. In a clinical setting, EphA2 inhibitors could be incorporated in ovarian cancer treatment strategies to disrupt tumor‐stroma interactions and enhance the efficacy of chemotherapy and immunotherapy. However, given the dual role of EphA2 in various cancers, precise modulation of EphA2 activity and specific delivery of inhibitors are essential to avoid off‐target effects.

## 5 Conclusion

To our knowledge, this is the first study to reveal the role of primary cilia in EndMT in the ovarian TME. Loss of primary cilia in ovarian TAEs induces EndMT via the EphA2 signaling pathway (Fig. 4H). As EndMT plays a vital role in ovarian cancer progression and metastasis, the enforced formation of primary cilia in ovarian TAEs may be a new strategy to cure chemoresistant ovarian cancer. However, further investigation is required to determine how cancer cells lose their primary cilia and how this enhances EphA2‐mediated EndMT.

## Conflict of interest

The authors declare no conflicts of interest.

## Author contributions

YY, JK, BSK, and JHP designed and supervised the study. JGC, YH, HIK, EY, AL, SH, DL, and SWK carried out experiments and analyzed the data. JGC and YH wrote the original draft. YY, JK, BSK, HIK, and SH reviewed and edited the manuscript. JHP, YY, JK, and BSK contributed to the discussion. All authors have read and approved the article.

## Supporting information


**Fig. S1.** Endothelial‐specific knockout of the *Kif3a* gene. PCR products were obtained after amplification of genomic DNA from the lungs, liver, and kidney of *Kif3a* ^*fl/fl*^ mice and *Cdh5‐Cre; Kif3a* ^*fl/fl*^ mice. A deletion indicates a recombined allele.

**Fig. S2.** Quantification of EphA2 expression in HUVECs. Three different si*EphA2* were used in this study. HUVECs were transfected with si*EphA2* 20 ng·L^−1^ for 48 h, and siGFP was used as a negative control. Cell lysates were analyzed by immunoblotting, and actin was used as a loading control.

## Data Availability

The data generated and/or analyzed during this study are available from the corresponding author upon a reasonable request.
