# Supplementary figures and images for: Loss of primary cilia promotes EphA2‐mediated endothelial‐to‐mesenchymal transition in the ovarian tumor microenvironment
Source: Mol Oncol. 2025 May 21;19(10):2951–66. doi: 10.1002/1878-0261.70057 (PMC12515696; doi:10.1002/1878-0261.70057)

A

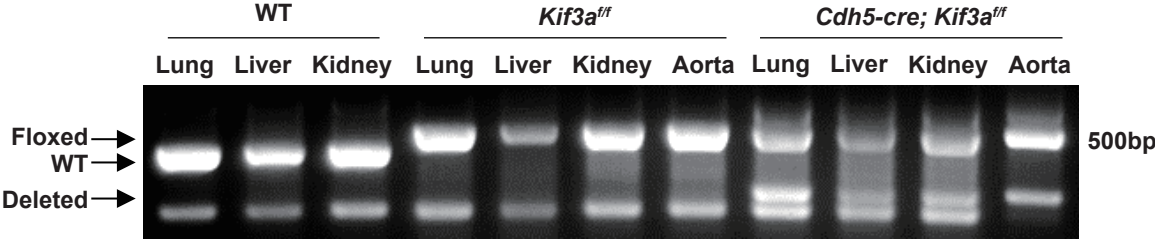

Supplement: Supplementary file 1 — Fig. S1. Endothelial‐specific knockout of the Kif3a gene. PCR products were obtained after amplification of genomic DNA from the lungs, liver, and kidney of Kif3a fl/fl mice and Cdh5‐Cre; Kif3a fl/fl mice. A deletion indicates a recombined allele. [file MOL2-19-2951-s001.pdf]

**A**

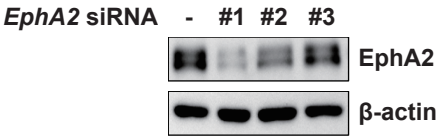

**B**

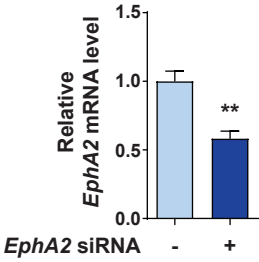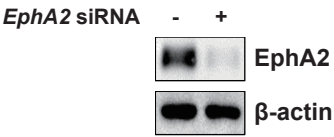

Supplement: Supplementary file 2 — Fig. S2. Quantification of EphA2 expression in HUVECs. Three different siEphA2 were used in this study. HUVECs were transfected with siEphA2 20 ng·L−1 for 48 h, and siGFP was used as a negative control. Cell lysates were analyzed by immunoblotting, and actin was used as a loading control. [file MOL2-19-2951-s002.pdf]
